# Development of simple random mutagenesis protocol for the protein expression system in *Pichia pastoris*

**DOI:** 10.1186/s13068-016-0613-z

**Published:** 2016-09-19

**Authors:** Mikako Tachioka, Naohisa Sugimoto, Akihiko Nakamura, Naoki Sunagawa, Takuya Ishida, Taku Uchiyama, Kiyohiko Igarashi, Masahiro Samejima

**Affiliations:** 1 Department of Biomaterial Sciences, Graduate School of Agricultural and Life Sciences, The University of Tokyo, Yayoi 1-1-1, Bunkyo-ku, Tokyo, 113-8657 Japan; 2 Biomaterial in Tokyo Co., Ltd., Fukuoka Lab, Ōnojō, Fukuoka 816-0905 Japan; 3 Institute for Molecular Science, National Institute of Natural Sciences, Okazaki, 444-8787 Japan

**Keywords:** Random mutagenesis, Cellulase, *Pichia pastoris*, Phi29 DNA polymerase, Error-prone RCA

## Abstract

**Background:**

Random mutagenesis is a powerful technique to obtain mutant proteins with different properties from the wild-type molecule. Error-prone PCR is often employed for random mutagenesis in bacterial protein expression systems, but has rarely been used in the methylotrophic yeast *Pichia pastoris* system, despite its significant advantages, mainly because large (μg-level) amounts of plasmids are required for transformation.

**Results:**

We developed a quick and easy technique for random mutagenesis in *P. pastoris* by sequential Phi29 DNA polymerase-based amplification methods, error-prone rolling circle amplification (RCA) and multiple displacement amplification (MDA). The methodology was validated by applying it for random mutation of the gene encoding cellulase from the basidiomycete *Phanerochaete chrysosporium* (*Pc*Cel6A), a key enzyme in degradation of cellulosic biomass. In the error-prone RCA step, the concentrations of manganese ion (Mn^2+^) and cellulase gene-containing plasmid were varied, and the products obtained under each condition were subjected to the second MDA step in the absence of Mn^2+^. The maximum error rate was 2.6 mutations/kb, as evaluated from the results of large-scale sequencing. Several μg of MDA products was transformed by electroporation into *Pichia* cells, and the activities of extracellularly expressed *Pc*Cel6A mutants towards crystalline and amorphous celluloses were compared with those of wild-type enzyme to identify key amino acid residues affecting degradation of crystalline cellulose.

**Conclusions:**

We present a rapid and convenient random mutagenesis method that does not require laborious steps such as ligation, cloning, and synthesis of specific primers. This method was successfully applied to the protein expression system in *P. pastoris*.

**Electronic supplementary material:**

The online version of this article (doi:10.1186/s13068-016-0613-z) contains supplementary material, which is available to authorized users.

## Background

Mutagenesis is an important technique in protein engineering to modify properties such as thermostability, optimum pH and specific activity, or to understand structure–function relationships of target proteins. There are two main approaches, i.e., rational design and random mutagenesis. Rational design is attractive when sufficient information is available about the protein structure, for example, if the key active-site amino acid residue(s) have been identified, even if their function is not fully established. On the other hand, random mutagenesis, i.e., introducing random mutations into the protein-coding gene and then screening the expressed mutant proteins, provides access to proteins having altered properties without human bias. Many proteins have been successfully engineered by means of random mutagenesis methods [1].

It is important that random mutagenesis methodology should be simple, because it is often necessary to screen large numbers of mutants, in contrast to the case of rational design. *Escherichia coli* and *Saccharomyces cerevisiae* are generally used as protein expression hosts for random mutagenesis because of the availability of various plasmids. However, heterologous expression of eukaryotic genes is often restricted in these organisms [2–4]. The methylotrophic yeast *Pichia pastoris* is a useful alternative, as it can perform many of the post-translational modifications found in higher eukaryotic cells, and it has been used to express proteins from a variety of different organisms, including human, vertebrates, fungi, plants and bacteria, in milligram-to-gram quantities [5]. Furthermore, *P. pastoris* is able to secrete heterologous proteins directly into the culture medium, which enables easy screening of mutant libraries and simplifies downstream purification. Despite these advantages, however, *P. pastoris* has rarely been used in random mutagenesis [2], mainly because the transformation efficiency is usually several orders of magnitude lower than that for *E. coli* and other yeasts [6]. Microgram amounts of plasmids are required for integration into the *P. pastoris* genome via homologous recombination, and therefore expression plasmids are often designed as shuttle vectors to enable rapid amplification in bacterial cloning hosts [7]. This technique is feasible for low-throughput analyses, but is too time-consuming for high-throughput experiments because of the need for thousands of bacterial transformations and plasmid isolations. To date, there are only a few established methods of random mutagenesis in this host [8–11].

Cellulase plays a key role in producing fermentable glucose from lignocellulosic biomass by hydrolyzing glycosidic bonds of cellulose, a linear biopolymer of β-1,4-linked d-glucose units. Cellulase has recently attracted much attention because of its enormous potential for application in commercial-scale biofuel production. However, the slow hydrolysis rate is still a bottleneck of this technology, because cellulose, especially crystalline cellulose, is quite resistant to hydrolysis. Therefore, improvement of cellulase performance has been a focus of research for many years and many researchers have attempted to engineer cellulase to improve the enzyme activity, thermal stability, or pH range [12–16]. Error-prone PCR and DNA shuffling have been used successfully in random mutagenesis of cellulases, but the limited host range for expression remains a key issue, especially for fungal cellulases. Other challenges include the complexity of transformation and screening operations with yeast or other eukaryotic host organisms, and low productivity of recombinant proteins. We have reported heterologous expression of many biomass utilization-related enzymes from the basidiomycete *Phanerochaete chrysosporium* in *P. pastoris* at the gram per liter of culture level [17–19]. In addition, utilization of *P. pastoris* for cellulase production would be advantageous for biofuel production, because this organism is able to ferment glucose to ethanol, and therefore it could be used in a consolidated bioprocess (CBP) involving simultaneous production of enzyme protein for sugar formation and fermentation of the sugar to obtain ethanol.

**Fig. 1:**
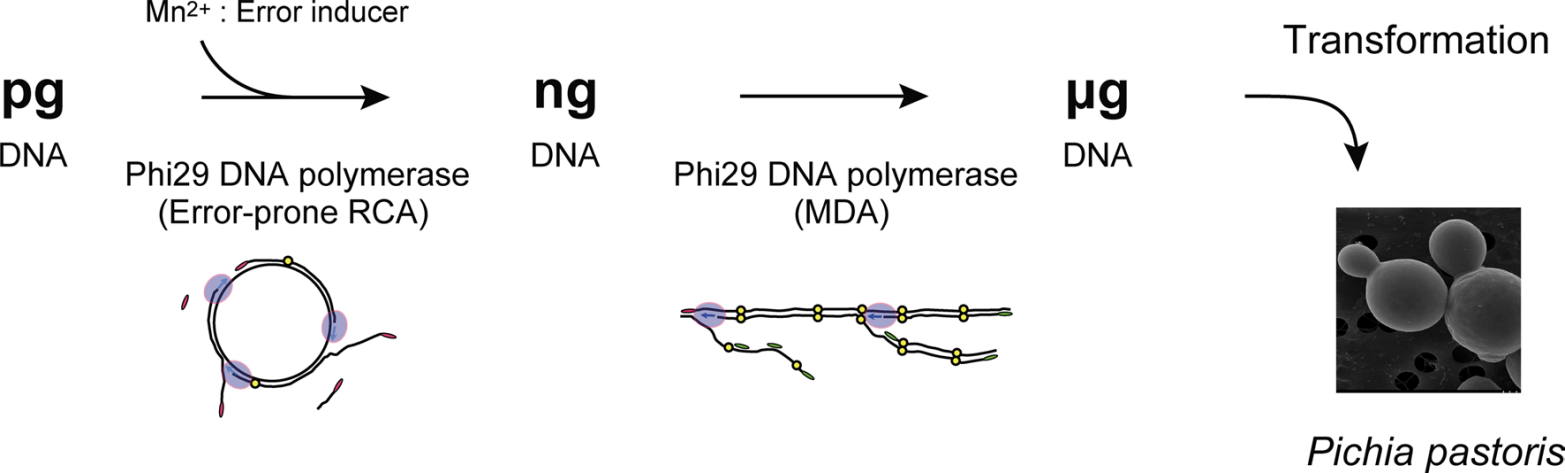
Schematic representation of the random mutagenesis method. The circular protein expression vector is amplified repeatedly by strand displacement reaction of Phi29 DNA polymerase. Mutations are introduced in the first step by adding Mn^2+^ to reduce the fidelity of the polymerase (this is known as error-prone RCA). Subsequent amplification with Phi29 DNA polymerase (MDA) provides μg amounts of mutated DNA, sufficient for transformation into *P. pastoris* for enzyme production

We describe here a simple random mutagenesis method for the *P. pastoris* expression system. To obtain microgram amounts of randomly mutated DNA, Phi29 DNA polymerase was repeatedly used (Fig. 1). This polymerase has strong strand displacement activity, and has been used for exponential amplification of circular DNA or genomic DNA [20–22]. The first step in our method is introduction of random mutations using the error-prone rolling circle amplification (RCA) method reported by Fujii [23, 24]. The second step is amplification of mutated DNA by Phi29 DNA polymerase, which is generally called multiple displacement amplification (MDA). The combination of these methods provide sufficient DNA to enable the *P. pastoris* expression system to be used easily for random mutagenesis experiments.

## Methods

### Materials

*Pichia pastoris* KM71H strain and the expression vector pGAPZαA were purchased from Invitrogen (Carlsbad, CA, USA). TempliPhi 100 DNA amplification kit was purchased from GE Healthcare (Buckinghamshire, UK). RepliPHI™ Phi29 Reagent Set and thiophosphate-modified random hexamer primer were bought from Epicentre (Madison, WI, USA) and Sigma-Aldrich (St. Louis, MO, USA), respectively. The restriction enzyme *Bln*I was purchased from TaKaRa (Shiga, Japan). Amorphous cellulose, phosphoric acid-swollen cellulose (PASC), was prepared from Avicel cellulose powder Funacel II from Funakoshi Ltd (Tokyo, Japan) [25]. Crystalline cellulose III_I_ was prepared from green algae *Cladophora* spp. as described previously [26]. Novozyme 188 β-glucosidase and Glucose CII Test Wako were purchased from Novozymes (Bagsværd, Denmark) and Wako Pure Chemical Industries (Osaka, Japan), respectively. MightyPrep reagent for DNA and KOD plus version 2 DNA polymerase were bought from TaKaRa (Shiga, Japan) and Toyobo (Osaka, Japan), respectively.

### Error-prone rolling circle amplification

The gene encoding *Pc*Cel6A from *P. chrysosporium* (*cel6A*) was cloned into the pGAPZαA vector based on the previous report [17]. The purified pGAPZα/*cel6A* vector was dissolved in water at the concentration of 100, 200, 500 or 1000 pg/μl and used as a template for the RCA reaction. RCA was performed using the Templiphi 100 DNA amplification kit based on the method described by Fujii et al. [23, 24]. In brief, 0.5 μl of the template was mixed with 5 μl of sample buffer containing random hexamers, and the mixture was heated at 95 °C for 3 minutes to denature the plasmid, and then immediately cooled to room temperature. The amplification reaction was started by addition of 1 μl of MnCl_2_ solution (0, 20, 40 mM), 5 μl of reaction buffer and 0.2 μl of enzyme mix. After 24-hours incubation at 30 °C, the mixture was heated at 65 °C for 10 minutes to inactivate the enzyme, and the amplification of DNA was confirmed by electrophoresis in a 0.8 % agarose gel. To estimate the amount of amplified vectors, the error-prone RCA product was treated with restriction enzyme *Bln*I for 3 hours and then subjected to 1 % agarose gel electrophoresis.

### Multiple displacement amplification of the error-prone RCA product

After error-prone RCA under 12 conditions (0, 1.0, 2.0 mM Mn^2+^ versus 50, 100, 250, and 500 pg of template), the second amplification by Phi29 DNA polymerase was conducted with the RepliPHI™ Phi29 Reagent Set. The error-prone RCA product was diluted 10 times with water and a 2.5 μl aliquot of the diluted product was mixed with 5 μl of 10× reaction buffer and 5 μl of 100 μM random hexamer. The mixture was heated at 95 °C for 3 minutes, then cooled to room temperature, and the amplification reaction was started by adding dNTPs, DTT and Phi29 DNA polymerase (total volume 50 μl). The final concentrations were 2 U/μl of Phi29 DNA polymerase, 10 μM of random hexamers, 5 mM DTT, and 0.25 mM each dNTP in 40 mM Tris–HCl buffer (pH 7.5) containing 10 mM MgCl_2_, 5 mM (NH_4_)_2_SO_4_ and 50 mM KCl. After incubation at 30 °C for 18 hours, the mixture was heated at 65 °C for 10 minutes to inactivate the enzyme and DNA amplification was confirmed by electrophoresis in 0.8 % agarose gel. To estimate the amount of amplified plasmids in the total amplified DNA, the product was digested with restriction enzyme *Bln*I for 3 hours and subjected to 1 % agarose gel electrophoresis.

### Large-scale sequencing of a mutant library

Twelve samples of MDA products obtained above were purified with a MinElute PCR purification kit (QIAGEN, Hilden, Denmark) after digestion with restriction enzyme *Bln*I. Sequencing was performed using an Illumina HiSeq 2000 according to the standard protocol of Illumina Inc. Briefly, library construction was performed according to the Illumina TruSeq™ DNA Sample Preparation Guide. Cluster generation took place on the Illumina cBot by using a TruSeq™ Rapid PE Cluster Kit. The libraries were then subjected to Illumina HiSeq 2000 sequencing according to standard procedures. Paired-end 100-bp reads were generated and the raw sequencing data was processed with the Illumina analysis pipeline (CASAVA ver. 1.8). Short reads were filtered and trimmed with the qtrim program (Genaris, Inc., Yokohama, Japan) to increase analytical precision. These short reads were aligned to the sequence data of pGAPZα/*cel6A* (4.4 kbp) using the Burroughs–Wheeler Aligner (BWA; Cambridge, UK) with default parameter settings.

### Mutation frequency analysis

The sequence data was analyzed to compare the mutation frequencies obtained under various conditions of error-prone RCA. Mutation frequency was calculated at each reference base of *cel6A* (1320 bp, A:255 T:256 G:330 C:479) by dividing the number of mismatches (substitutions, insertions and deletions) by the number of total sequenced bases. A histogram was generated with IGOR Pro software (Ver. 6.1) by plotting mutation frequency at each reference base. Curve fittings were performed by using the log-normal distribution of Multi-peak fit function (Ver. 2.00). Heat maps were drawn by Microsoft Excel. The GC content was defined as the percentage of GC in 6 neighbor sequences. The heat map was also drawn for the whole vector pGAPZα/*cel6A* in a similar way with the *cel6A* region analysis.

### Production of enzymes in *Pichia pastoris*

Approximately 5 μg of the MDA product (50 μl of the MDA product) was digested with *Bln*I prior to transformation of *P. pastoris*. After purification of DNA by ethanol precipitation, electroporation was carried out according to the instruction manual of the EasySelect™ *Pichia* expression kit. The plasmids were also prepared by the usual preparation method described in the manual, including *E. coli* transformation and plasmid isolation by miniprep, and were transformed as a control. The transformed cells were plated in YPDS agar (1 % yeast extract, 2 % polypeptone, 2 % glucose, 1 M sorbitol, 1.5 % agar) plus zeocin (100 μg/ml) and incubated at 30 °C. Single colonies of the transformants were transferred into deep-well microtiter plates containing 1 ml of YPDZ media per well (1 % yeast extract, 2 % polypeptone, 2 % glucose, 100 μg/ml zeocin) and incubated at 30 °C for 3 days with shaking at 1400 rpm. Gene expression was driven by the constitutive GAP promoter, and the protein was accumulated during 3 days cultivation without addition of any inducer.

### Characterization of mutant enzymes

All hydrolysis experiments were performed in 96-well Multiscreen HTS plates (Millipore), of which the bottoms were sealed. Ten μl aliquots of yeast culture supernatants were incubated with PASC (0.05 %, w/v) or cellulose III_I_ (0.05 %, w/v) for 2 hours at 40 °C with shaking at 1000 rpm. Each well contained 100 mM sodium acetate buffer (pH 5.0), and Novozyme 188 β-glucosidase was added to the mixture to convert cellooligosaccharides to glucose. The mixture was filtered through the plates to terminate the reaction. The amount of released sugars was determined using Glucose CII Test Wako: 50 μl of filtrate was mixed with 150 μl of the test kit and the absorbance of 492 nm was measured with a Multiskan FC (Thermo Fisher Scientific, USA).

The PASC-degrading activity was measured for 87 transformants obtained by the error-prone RCA-MDA under the conditions of 2 mM Mn^2+^ and 100 pg template, 100 transformants under the conditions of 2 mM Mn^2+^and 250 pg template, and 96 colonies obtained by the usual plasmid preparation method. For further characterization, 87 transformants whose activity was more than 10 % of the wild-type activity were randomly selected from those obtained by error-prone RCA-MDA listed above. The PASC- and cellulose III_I_-degrading activities were measured for all 87 selected colonies and the *cel6A* region was sequenced as follows. Colonies on the plate were picked up, suspended in 100 µl MightyPrep reagent and incubated at 95 °C for 10 minutes for DNA extraction. PCR were performed using KOD plus DNA polymerase. The PCR products were purified and the inserted *cel6A* region was sequenced using the Eurofins sequencing service (Eurofins MWG Operon). Total protein concentration of each culture supernatant was determined by Bradford assay (Bio-rad, Hercules CA, USA) with bovine serum albumin (BSA) as a standard. The three-dimensional structure of *Pc*Cel6A catalytic domain was determined (submitted elsewhere) and the structure of CBM was predicted by using Protein Homology/analogy Recognition Engine (Phyre) version 2.0 (http://www.sbg.bio.ic.ac.uk/phyre2/). Figures for molecular models were prepared using the PyMOL Molecular Graphics System, Version 1.7 Schrödinger, LLC [27].

## Results and discussion

We have developed a novel, rapid random mutagenesis strategy that enables the *P. pastoris* expression system to be used for directed evolution of eukaryotic proteins, despite its low transformation efficiency. This method is composed of sequential plasmid amplification by means of error-prone RCA and MDA of the error-prone RCA products, followed by protein expression in *P. pastoris* (Fig. 1).

### Error-prone rolling circle amplification

Phi29 DNA polymerase amplifies circular DNAs isothermally and yields linear DNAs composed of tandem repeats of the circular DNA plasmids [28]. In error-prone RCA, the gene coding a target protein is first cloned into an expression vector and then the whole vector is amplified in the presence of Mn^2+^ to introduce mutations of the target gene [23, 24]. In this study, various amounts of circular pGAPZα vectors including *cel6A* gene (50–500 pg) were amplified in the presence of 0–2 mM MnCl_2_.

**Fig. 2:**
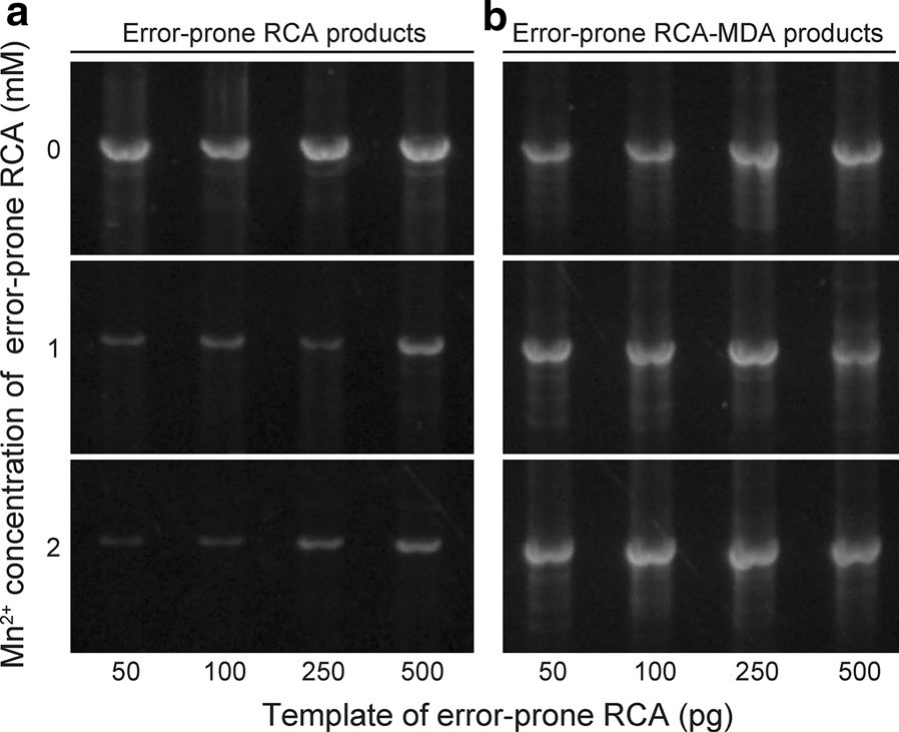
Yield of the error-prone RCA and MDA products after digestion with restriction enzyme. Various amounts of plasmid (pGAPZα/*cel6A*) were amplified with MnCl_2_ (**a**), and the obtained error-prone RCA products were amplified without MnCl_2_ (**b**). Amplified DNAs were digested with restriction enzyme BlnI, which cleaves a single site in the vector. The DNA separated by agarose gel electrophoresis showed a single plasmid-sized band (4.4 kb)

The products thus obtained appeared at positions corresponding to more than 10 kbp in agarose gel electrophoresis. Therefore, the products were digested with restriction enzyme (*Bln*I) to afford linear fragments of 4.4 kbp, which is identical to the sum of vector and insert. The yield of the products decreased with increase of Mn^2+^ concentration and also with decrease of the initial amount of template, as shown in Fig. 2a. The largest amount of amplified plasmids estimated from gel electrophoresis was ~1 μg in a total volume of 10 μl. However, plasmids obtained in the presence of 2 mM Mn^2+^ were less than 10 ng/µl, and no band of the expected size appeared in the presence of 4 mM Mn^2+^ (data not shown).

The amount of the error-prone RCA products shown in Fig. 2a is more than enough for transformation in the *E. coli* expression system, as reported by Fujii and coworkers [23]. *E. coli* needs only a ng-level amount of plasmids for transformation, but on the other hand, it is not enough for the *Pichia* expression system. The transformation efficiency of *E. coli* is generally 10^8^–10^11^ transformants per µg of DNA, on the other hand, the electroporation of *P. pastoris* yields 10^3^–10^4^ transformants per µg of linearized DNA [6, 29]. The low transformation efficiency of *P. pastoris* is unavoidable because the yeast cell has thicker walls compared with Bacteria and plasmid must enter through the walls and be integrated into specific locations in the chromosome. The difference in the vector integration results in the increased stability of expression strains but the reduced transformation efficiency.

### Multiple displacement amplification of the error-prone RCA product

To obtain plasmids with random mutation on a larger (μg) scale, we used Phi29 DNA polymerase again for amplification of the linear DNA by means of MDA. Twelve samples of the error-prone RCA product (0, 1, 2 mM Mn^2+^ versus 50, 100, 250, 500 pg of template) were diluted 10-fold and directly used as templates for MDA amplification. As shown in Fig. 2b, almost the same level of amplification was obtained for all samples, indicating that the initial RCA products are long enough to serve as templates of MDA. The amount of linear plasmids obtained after restriction enzyme treatment was about 5 μg in a total volume of 50 μl, which is enough for *P. pastoris* transformation.

### Analysis of mutation frequency using large-scale sequencing

To evaluate the mutation frequency after the two-step amplification, the MDA products were sequenced with an Illumina sequencer. The number of sequenced bases for each error-prone RCA condition was 0.6–4 gb/sample for the 4.4 kbp vector (Additional file 1: Table S1). After removal of low-quality bases, we analyzed errors in the *cel6A* region. As we used large-scale sequencing, we could calculate the mutation frequency at each base (*cel6A* 1320 bp, A:255 T:256 G:330 C:479) for detailed analysis.

**Fig. 3:**
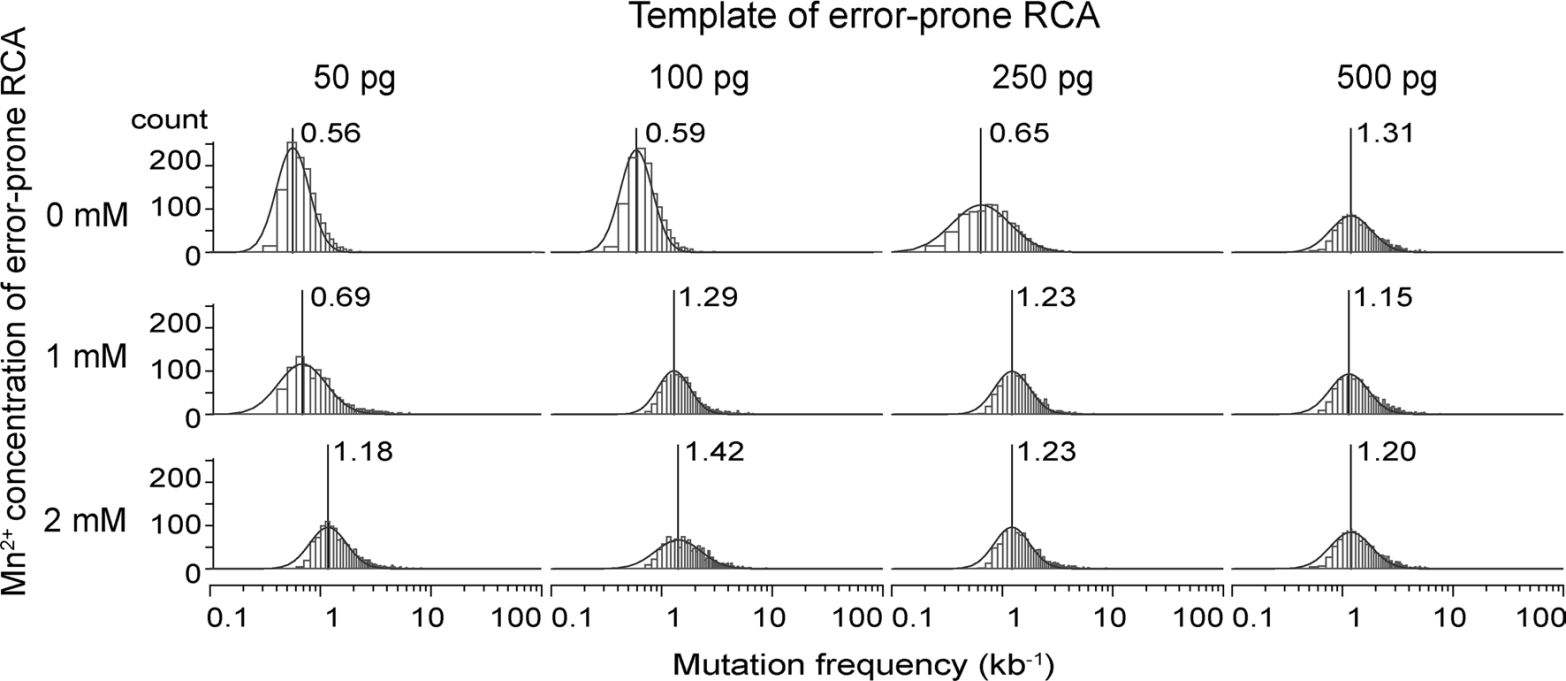
Histogram of per-base mutation frequency on a logarithmic scale. The distributions of mutation frequency in each reference position of the *Pc*Cel6A gene were fitted to a log-normal distribution (*solid line*). Peak locations are shown with *vertical bars*

The minimum and maximum of total sequenced bases at each base of *cel6A* and the averaged mutation frequencies are shown in Table 1. The maximum mutation frequency (2.60 kb^−1^) was obtained with 2 mM Mn^2+^ and 100 pg template. The mode and distribution of mutation frequency were also analyzed and the results are shown as a histogram in Fig. 3. Under all conditions, the bases of *cel6A* were log-normally distributed. The number of errors increased with increasing concentration of Mn^2+^ in accordance with Fujii’s findings [23]. Surprisingly, at high template concentration (500 pg), the averaged mutation frequency was high (2.07 kb^−1^) even in the absence of Mn^2+^ in the reaction mixture. Thus, higher concentrations of Mn^2+^ and templates tended to result in higher error ratios.

**Table 1:** Mutation frequency

Template (pg)	Mn^2+^ (mM)	Total bases^a^		Averaged mutation frequency (kb^−1^)
		Minimum	Maximum	
50	0	452,854	697,971	0.85 ± 0.56
	1	768,351	1,250,127	1.55 ± 1.40
	2	394,019	782,970	1.98 ± 1.76
100	0	459,038	721,539	0.86 ± 0.55
	1	112,234	169,952	2.15 ± 2.08
	2	414,024	782,275	2.60 ± 4.63
250	0	141,208	223,967	1.32 ± 1.38
	1	272,842	419,288	1.98 ± 1.92
	2	220,309	433,775	2.03 ± 1.84
500	0	186,120	294,496	2.07 ± 1.95
	1	213,880	463,802	1.99 ± 1.76
	2	184,115	406,056	2.11 ± 1.92

^a^The total number of bases used for calculation of mutation frequency at each reference base of *cel6A* (1320 bp)

The types of substitution mutation varied, as shown in Additional file 1: Tables S2–S5. The mutation frequency of each substitution is shown on the left side of each column to illustrate any inherent bias of this error-prone RCA-MDA method, and the proportion of the total mutations in *cel6A* is shown in parentheses. The exchange of A to C revealed an overall trend towards high mutation frequency, reaching 1.44–1.65 kb^−1^ under 9 conditions. The transition/transversion ratio was 0.4–0.6, except for the condition with 1 mM Mn^2+^ and 50 pg templates. These results are different from Fujii’s findings, in which mutations were strongly biased in favour of C to T and G to A (66 %) and the transition/transversion ratio was 2.7 [23]. The distribution of mutations is analysed and shown in relation to GC content at each base of *cel6A* (Additional file 1: Figure S1). There were some regions where mutations continuously appeared (positions 770–830, 1000–1050), indicating that errors were more frequently distributed in GC-rich regions (Additional file 1: Figures S2–S3). The mutations were also found in the whole vector regions including promoter regions and selection marker gene (Additional file 1: Figure S4), which potentially affect the transformation efficiency or protein productivity.

### Transformation, enzyme production, and measurements of cellulase activities

Error-prone RCA-MDA products obtained under two conditions (2 mM manganese and 100, 250 pg template) were successfully transformed into *P. pastoris* after restriction enzyme digestion. The number of colonies was ~100 per plate, which is slightly fewer than the plates of wild-type colonies obtained by the usual plasmids preparation protocol (100–300 colonies per plate).

**Fig. 4:**
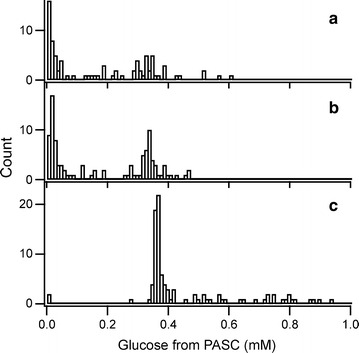
Histogram of amorphous cellulose (PASC)-degrading activities of transformants. **a** The activities of 87 transformants obtained by error-prone RCA-MDA under the conditions of 2 mM Mn^2+^ and 100 pg template. **b** The activities of 100 transformants obtained by error-prone RCA-MDA under the conditions of 2 mM Mn^2+^and 250 pg template. **c** The activities of 96 transformants obtained by the usual plasmids preparation protocol (wild-type control)

All colonies from error-prone RCA-MDA plates, excluding small colonies, and 96 colonies from the control plate were incubated in liquid culture for cellulase production. Then the amorphous cellulose (PASC)-degrading activity of the crude enzymes was measured, and the results obtained for the transformants of error-prone RCA-MDA are shown with those of wild-type in Fig. 4. The activity of wild-type transformants were dispersed (Fig. 4c), indicating that the activities would be influenced by differing amounts of enzymes in the culture resulting from the different productivity of the enzymes. Especially, multiple gene integration events occur with detectable frequency and greatly enhance the expression level of a target protein [30, 31]. The numbers of transformants whose activities were less than 10 % of that of wild-type *Pc*Cel6A (the median activity of 96 control transformants was 0.37 mM glucose in 2-hour incubation) were 40 and 37 % under the conditions with 100 and 250 pg template, respectively (Fig. 4a, b). In contrast, the corresponding number for wild-type *Pc*Cel6A was 2 % (2 of 96 colonies, Fig. 4c). We consider that the high levels of transformants with markedly lowered activity from the error-prone RCA-MDA plates are mainly due to the introduction of mutations.

### Degradation of amorphous and crystalline celluloses by mutants

**Fig. 5:**
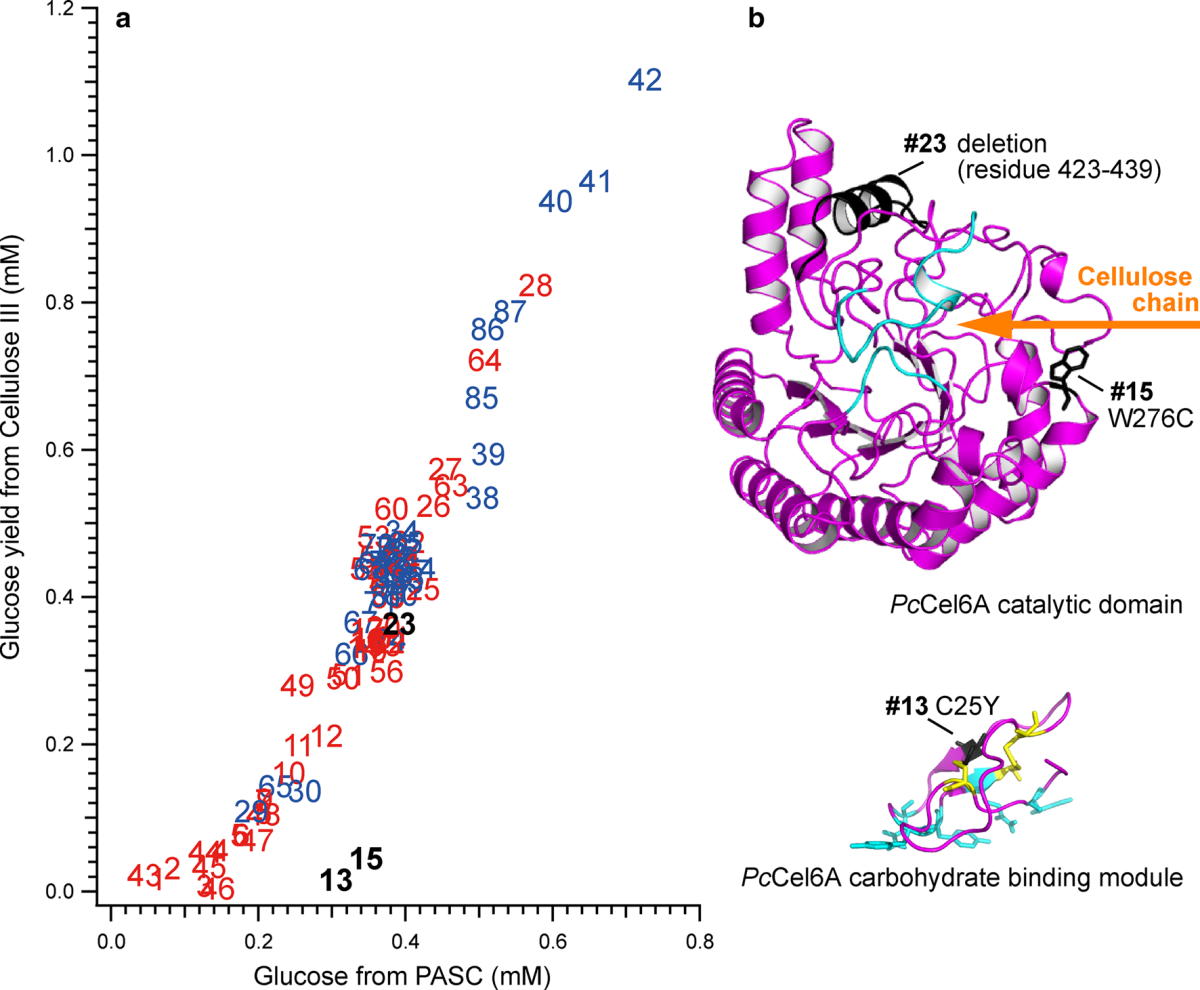
**a** Plot of amorphous cellulose (PASC)-degrading activity versus crystalline cellulose (cellulose III_I_)-degrading activity of *Pc*Cel6A mutants. Forty-two transformants were selected from error-prone RCA-MDA plates under the conditions of 2 mM Mn^2+^ and 100 pg template (numbered 1–42), and 45 transformants from error-prone RCA-MDA plates under the conditions of 2 mM Mn^2+^ and 250 pg template (numbered 43–87), and their activities were measured. The transformants with no mutation or no change in amino acid sequence are indicated in* blue*. Mutants with at least one mutation in the *cel6A* gene are shown in* red*. Mutants discussed in the text are shown in *black*. **b** The mutations found in mutants #13, #15, and #23. The structures of *Pc*Cel6A catalytic domain and CBM were modeled and the locations of altered amino acids are indicated with the mutant numbers. The active site loops of the catalytic domain are colored in* cyan* and the direction of the incoming cellulose chain is indicated by an *orange arrow*. Two disulfide bridges in CBM are colored in *yellow*

Next, we compared the activities of the crude mutant enzymes to degrade amorphous and crystalline celluloses. Figure 5a shows the amorphous cellulose (PASC)-degrading versus crystalline cellulose (cellulose III_I_)-degrading activities of 87 randomly selected transformants from error-prone RCA-MDA plates, which had activities of more than 10 % of that of the wild type in Fig. 4. The data points in blue in Fig. 5a shows the transformants for which DNA sequencing revealed no mutation or no change in amino acid sequence. Mutants that had at least one mutation in the *cel6A* gene are shown in red in Fig. 5a. The activities of these mutants were affected by relatively large difference in protein expression levels, as the total protein concentrations of culture supernatants were varied from 0.01 to 0.2 mg/ml. The results of SDS-PAGE analysis of some mutants were shown in Additional file 1: Figure S5.

Most of the mutants had an amorphous/crystalline cellulose-degrading activity ratio similar to that of the wild type, though two mutants revealed a clearly different character. Mutants #13 and #15 had lowered degrading activity towards crystalline cellulose III_I_ while retaining activity towards amorphous cellulose. The DNA sequencing of #15 revealed a single mutation, W267C. This tryptophan residue is located at the entrance of the active site tunnel of *Pc*Cel6A (Fig. 5b), and the importance of the residue has been demonstrated in *Trichoderma reesei* Cel6A: it is not necessary for hydrolysis, but is requisite for loading a cellulose chain from the crystalline surface [32]. On the other hand, the DNA sequencing of #13 revealed several mutations: C25Y, A105D, G346D, as well as two mutations that would not cause any amino acid substitution. The most influential mutation is probably C25Y, because C25 is expected to form a disulfide bridge with C8 in the carbohydrate-binding module (CBM) of *Pc*Cel6A (Fig. 5b), and this mutation is expected to result in reduced affinity and adsorption on the crystalline surface [33]. However, the DNA sequencing traces of #13 showed overlaps with the wild-type sequence, probably due to multiple-copy gene integration, while the multiple integration event occurs less than 10 % of transformed colonies [34]. It might result in the production of different proteins in a single *Pichia* cell, and the activity would be a mixture of mutants. Interestingly, deletion of 17 amino acids at the C-terminus did not cause a significant change of activity, as seen in the case of mutant #23 with a stop codon at W423 and two silent mutations.

A major advantage of the *P. pastoris* expression system is that the enzyme is secreted extracellularly, so that the cellulase activity of *Pc*Cel6A mutants can be easily measured by the direct use of culture filtrates. In the present study, we found two mutants with altered crystalline cellulose degradation by *Pc*Cel6A in relatively small libraries, supporting the idea that *P. pastoris* is a useful system for screening of secreted proteins. The use of *P. pastoris* could be especially advantageous for screening of enzymes with insoluble substrates or substrates that are unable to diffuse through cell membranes. Moreover, by utilizing β-glucosidase-producing *P. pastoris*, direct screening in terms of growth difference might be possible.

## Conclusions

**Fig. 6:**
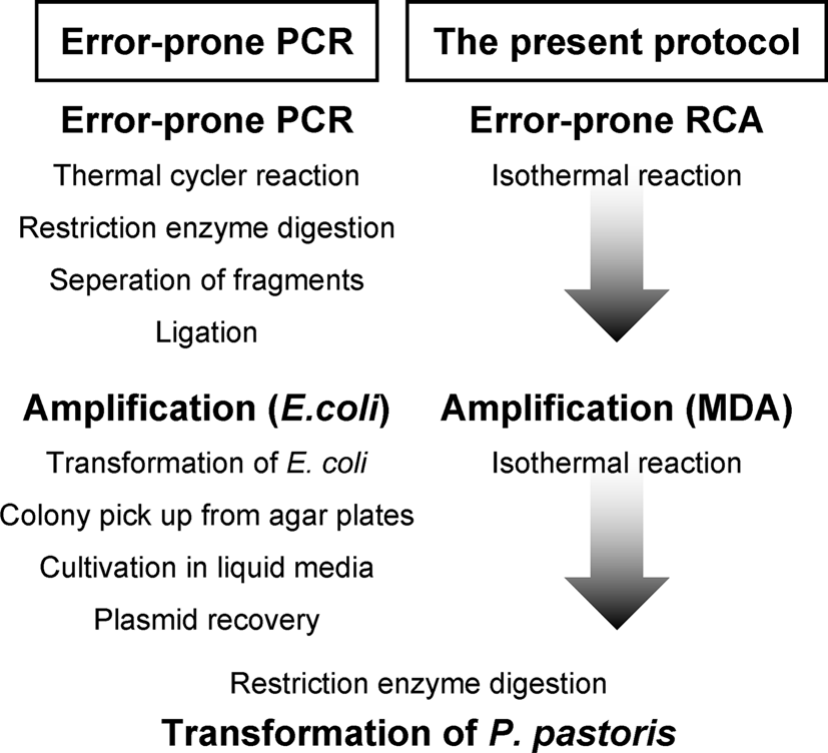
Comparison of random mutagenesis methods for the *P. pastoris* expression system

The goal of this study was to establish the suitability of *P. pastoris* as an expression system for random mutagenesis, because this organism has the capability to perform many eukaryotic post-translational modifications. Furthermore, *P. pastoris* is an excellent choice for the production of secreted proteins on account of its limited endogenous protein secretion. To obtain sufficient amounts of mutated DNA, we developed a rapid and convenient random mutagenesis method that does not require laborious steps such as ligation, cloning, and synthesis of specific primers (Fig. 6). This method uses widely available kits and takes the advantage of the fact that cloned DNA is typically obtained in circular vectors. Each experimental step takes only a few minutes, and microgram amounts of mutated DNA can be obtained within 3 days. Mutation frequencies were consistently in the range of 2–3 base substitutions per kb in the presence of 2 mM manganese ions, and this error rate is considered to be favorable for accumulation of adaptive mutations [35]. The prepared DNAs performed normally as vectors for transformation of *P. pastoris.* This method was successfully applied to the *P. pastoris* system, but should also be applicable to many other transformation systems that employ linear vectors.

## Additional file

10.1186/s13068-016-0613-z Additional Tables and Figures.

## Abbreviations

CBM: carbohydrate-binding module
MDA: multiple displacement amplification
PASC: phosphoric acid-swollen cellulose
RCA: rolling circle amplification
