# Supplementary material for: Development of simple random mutagenesis protocol for the protein expression system in Pichia pastoris
Source: Biotechnol Biofuels. 2016 Sep 19;9:199. doi: 10.1186/s13068-016-0613-z (PMC5028916; doi:10.1186/s13068-016-0613-z)
Supplement: Supplementary file 1 — 10.1186/s13068-016-0613-z Additional Tables and Figures. [file 13068_2016_613_MOESM1_ESM.pdf]

Additional supporting files

Development of simple random mutagenesis protocol for the protein expression system in *Pichia pastoris*

Mikako Tachioka, Naohisa Sugimoto[†], Akihiko Nakamura[‡], Naoki Sunagawa, Takuya Ishida, Taku Uchiyama, Kiyohiko Igarashi and Masahiro Samejima[*]

This PDF file includes: Tables, Figures

Tables

Table S1. Summary of Illumina sequencing data.

Template (pg)		50			100	
Mn^{2+} (mM)	0	1	2	0	1	2
Total reads	35,875,408	8,081,358	20,091,380	35,926,976	14,462,094	35,999,440
Average length	68.0	70.6	72.4	72.1	71.7	72.4
Total bases	2,440,638,535	570,099,258	1,453,831,528	2,588,644,474	1,036,793,040	2,606,169,302
P20 bases*	2,424,785,994	561,139,723	1,434,160,167	2,554,169,451	1,021,406,344	2,572,067,857
Average quality	37.9	36.8	37.0	37.0	36.8	37.0
Properly mapped reads	34,603,742	7,736,880	19,299,884	34,705,958	14,081,476	34,769,850

Template (pg)		250			500	
Mn^{2+} (mM)	0	1	2	0	1	2
Total reads	9,715,804	20,914,728	20,091,380	38,542,866	19,903,026	22,455,500
Average length	73.4	72.4	72.4	68.5	71.6	73.1
Total bases	713,374,274	1,514,941,723	1,453,831,528	2,640,727,717	1,424,643,642	1,641,096,327
P20 bases*	701,306,322	1,494,428,155	1,434,160,167	2,622,966,123	1,404,115,470	1,619,650,927
Average quality	36.2	37.0	37.0	37.9	36.8	37.1
Properly mapped reads	9,547,646	20,252,516	19,299,884	37,095,176	19,533,650	21,664,400

* P20 bases represents the total bases whose Phred Quality Scores were more than 2

Table S2. Averaged mutation frequency in *cel6A* (50 pg template). Averaged mutation frequency (numbers on left side of each column) was calculated by averaging the mutation frequency at each reference base. Percentage of total mutations in *cel6A* is shown in parentheses.

**Template (pg)**			**50**			
**Mn^{2+} (mM)**	**0**		**1**		**2**	
Total bases (Maximum)	697,971		1,250,127		782,970	
Total bases (Minimum)	162,764		250,735		11,583	
**Averaged mutation frequency (kb^{-1})**						
**Total (*cel6A*)**	**0.85 ± 0.56**		**1.55 ± 1.40**		**1.98 ± 1.76**	
**Transitions**	**0.31**	**(36%)**	**0.74**	**(52%)**	**0.66**	**(32%)**
A to G	0.30	(7%)	0.37	(5%)	0.69	(7%)
T to C	0.38	(9%)	0.40	(5%)	0.87	(9%)
G to A	0.30	(9%)	1.28	(21%)	0.57	(7%)
C to T	0.27	(12%)	0.91	(21%)	0.50	(9%)
**Transversions**	**0.26**	**(62%)**	**0.35**	**(47%)**	**0.70**	**(68%)**
A to T	0.23	(5%)	0.31	(4%)	0.49	(5%)
T to A	0.20	(5%)	0.24	(3%)	0.41	(4%)
A to C	0.35	(8%)	0.36	(5%)	1.47	(14%)
T to G	0.24	(6%)	0.26	(3%)	0.91	(9%)
G to C	0.21	(6%)	0.23	(4%)	0.51	(6%)
C to G	0.16	(7%)	0.17	(4%)	0.36	(7%)
G to T	0.36	(11%)	0.67	(11%)	0.74	(9%)
C to A	0.36	(15%)	0.59	(14%)	0.70	(13%)
**Insertions, Deletions**	**0.01**	**(2%)**	**0.02**	**(1%)**	**0.02**	**(1%)**
Transitions/Transversions	0.6	(0.6)	1.1	(1.1)	0.5	(0.5)
AT to GC/GC to AT	1.2	(0.8)	0.4	(0.2)	1.5	(0.9)
A to N, T to N	1.70	(39%)	1.94	(24%)	4.85	(47%)
G to N, C to N	1.08	(59%)	1.65	(74%)	2.32	(52%)

Table S3. Averaged mutation frequency in *cel6A* (100 pg template). These values were obtained according to the legend in Table S2.

**Template (pg)**	**100**					
**Mn^{2+} (mM)**	**0**		**1**		**2**	
Total bases (Maximum)	721,539		169,952		782,275	
Total bases (Minimum)	166,366		40,510		14,967	
**Averaged mutation frequency (kb^{-1})**						
**Total (*cel6A*)**	**0.86 ± 0.55**		**2.15 ± 2.08**		**2.60 ± 4.63**	
**Transitions**	**0.32**	**(37%)**	**0.77**	**(33%)**	**0.71**	**(26%)**
A to G	0.30	(7%)	0.80	(7%)	0.68	(5%)
T to C	0.39	(9%)	1.10	(10%)	0.87	(7%)
G to A	0.31	(9%)	0.68	(8%)	0.82	(8%)
C to T	0.28	(12%)	0.48	(8%)	0.46	(6%)
**Transversions**	**0.27**	**(62%)**	**0.77**	**(66%)**	**0.93**	**(72%)**
A to T	0.24	(5%)	0.69	(6%)	0.47	(4%)
T to A	0.21	(5%)	0.59	(5%)	0.63	(5%)
A to C	0.36	(8%)	1.64	(15%)	1.46	(11%)
T to G	0.25	(6%)	0.92	(8%)	0.92	(7%)
G to C	0.21	(6%)	0.52	(6%)	0.52	(5%)
C to G	0.15	(7%)	0.28	(5%)	0.37	(5%)
G to T	0.35	(10%)	0.76	(9%)	1.55	(15%)
C to A	0.35	(15%)	0.71	(12%)	1.53	(21%)
**Insertions, Deletions**	**0.01**	**(1%)**	**0.01**	**(0%)**	**0.04**	**(2%)**
Transitions/Transversions	0.6	(0.6)	0.5	(0.5)	0.4	(0.4)
AT to GC/GC to AT	1.2	(0.7)	1.6	(1.1)	1.2	(0.8)
A to N, T to N	1.76	(40%)	5.74	(52%)	5.04	(37%)
G to N, C to N	1.07	(59%)	2.28	(48%)	3.97	(61%)

Table S4. Averaged mutation frequency in *cel6A* (250 pg template). These values were obtained according to the legend in Table S2.

**Template (pg)**	**250**					
**Mn^{2+} (mM)**	**0**		**1**		**2**	
Total bases (Maximum)	223,967		419,288		433,775	
Total bases (Minimum)	45,307		105,653		6,415	
**Averaged mutation frequency (kb^{-1})**						
**Total (*cel6A*)**	**1.32 ± 1.38**		**1.98 ± 1.92**		**2.03 ± 1.84**	
**Transitions**	**0.46**	**(33%)**	**0.62**	**(29%)**	**0.63**	**(29%)**
A to G	0.45	(7%)	0.68	(7%)	0.70	(7%)
T to C	0.66	(10%)	0.97	(10%)	0.90	(9%)
G to A	0.41	(8%)	0.43	(5%)	0.49	(6%)
C to T	0.33	(9%)	0.39	(7%)	0.44	(8%)
**Transversions**	**0.45**	**(66%)**	**0.73**	**(71%)**	**0.74**	**(70%)**
A to T	0.43	(6%)	0.46	(4%)	0.50	(5%)
T to A	0.29	(4%)	0.37	(4%)	0.41	(4%)
A to C	0.84	(12%)	1.58	(15%)	1.53	(15%)
T to G	0.48	(7%)	0.89	(9%)	0.94	(9%)
G to C	0.23	(4%)	0.56	(7%)	0.53	(7%)
C to G	0.12	(3%)	0.34	(6%)	0.38	(7%)
G to T	0.61	(12%)	0.84	(11%)	0.81	(10%)
C to A	0.61	(17%)	0.81	(15%)	0.81	(14%)
**Insertions, Deletions**	**0.01**	**(1%)**	**0.01**	**(0%)**	**0.01**	**(1%)**
Transitions/Transversions	0.5	(0.5)	0.4	(0.4)	0.4	(0.4)
AT to GC/GC to AT	1.5	(1.0)	2.0	(1.3)	1.7	(1.1)
A to N, T to N	3.14	(46%)	4.95	(48%)	4.98	(48%)
G to N, C to N	1.57	(53%)	2.55	(51%)	2.53	(52%)

Table S5. Averaged mutation frequency in *cel6A* (500 pg template). These values were obtained according to the legend in Table S2.

**Template (pg)**			**500**			
**Mn^{2+} (mM)**	**0**		**1**		**2**	
Total bases (Maximum)	294,496		463,802		406,056	
Total bases (Minimum)	68,363		4,189		3,829	
**Averaged mutation frequency (kb^{-1})**						
**Total (*cel6A*)**	**2.07 ± 1.95**		**1.99 ± 1.76**		**2.11 ± 1.92**	
**Transitions**	**0.64**	**(28%)**	**0.63**	**(29%)**	**0.72**	**(33%)**
A to G	0.72	(7%)	0.70	(7%)	0.75	(7%)
T to C	1.00	(9%)	0.90	(9%)	0.94	(9%)
G to A	0.45	(5%)	0.48	(6%)	0.59	(7%)
C to T	0.39	(7%)	0.43	(8%)	0.60	(10%)
**Transversions**	**0.76**	**(71%)**	**0.73**	**(70%)**	**0.74**	**(67%)**
A to T	0.48	(4%)	0.50	(5%)	0.54	(5%)
T to A	0.39	(4%)	0.41	(4%)	0.43	(4%)
A to C	1.65	(15%)	1.44	(14%)	1.54	(14%)
T to G	0.93	(9%)	0.95	(9%)	0.96	(9%)
G to C	0.58	(7%)	0.54	(7%)	0.57	(7%)
C to G	0.37	(6%)	0.37	(7%)	0.39	(7%)
G to T	0.86	(10%)	0.82	(10%)	0.75	(9%)
C to A	0.85	(15%)	0.78	(14%)	0.72	(12%)
**Insertions, Deletions**	**0.01**	**(1%)**	**0.01**	**(1%)**	**0.02**	**(1%)**
Transitions/Transversions	0.4	(0.4)	0.4	(0.4)	0.5	(0.5)
AT to GC/GC to AT	2.0	(1.3)	1.7	(1.1)	1.4	(0.9)
A to N, T to N	5.17	(48%)	4.89	(48%)	5.15	(47%)
G to N, C to N	2.65	(51%)	2.51	(52%)	2.43	(52%)

Figures

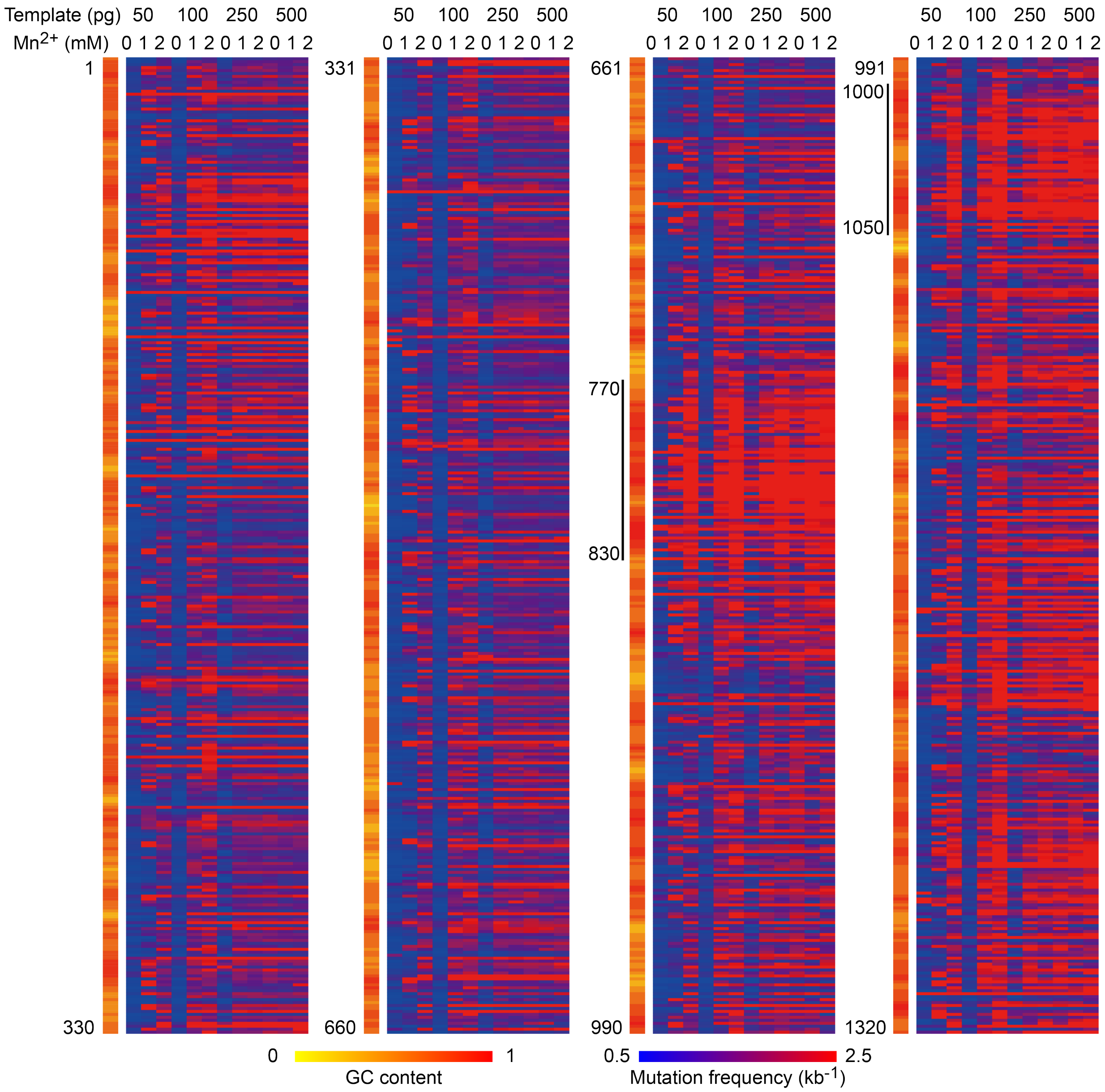

Fig. S1. Sequence dependency of mutation frequency. The mutation frequency of each base in *cel6A* (1320 bp) was calculated. The GC content was defined as the percentage of GC in 6 neighbor sequences.

Template (pg)			50			100			250			500		
Mn^{2+} (mM)			0	1	2	0	1	2	0	1	2	0	1	2
770	T	0.4	0.91	0.91	2.07	0.81	2.19	2.16	1.18	2.13	2.21	2.13	1.94	2.29
771	G	0.4	0.80	1.27	1.77	0.86	1.62	2.93	1.40	1.93	1.97	2.06	1.86	1.50
772	G	0.4	0.61	0.94	1.42	0.70	1.25	1.68	0.68	1.21	1.51	1.11	1.49	1.67
773	A	0.6	0.57	0.76	2.48	0.66	2.14	2.67	0.86	1.53	2.56	1.60	2.40	2.74
774	T	0.6	0.73	0.74	2.51	0.82	2.01	2.20	1.24	2.03	2.58	2.00	2.64	2.90
775	G	0.6	0.78	1.10	1.47	0.77	1.94	1.68	1.02	1.94	1.56	1.76	1.53	1.71
776	C	0.6	0.86	2.90	4.51	0.85	2.49	4.87	1.21	2.26	4.75	2.04	5.19	5.00
777	T	0.7	0.92	1.26	3.15	0.92	3.01	3.04	1.66	2.58	3.42	2.57	3.51	4.05
778	G	0.9	1.57	1.22	1.97	0.88	2.15	2.68	1.27	1.92	2.14	2.22	2.37	2.16
779	G	0.7	1.04	5.12	2.96	0.92	4.78	2.75	1.64	1.75	1.88	1.86	1.78	2.23
780	C	0.7	0.88	4.05	3.48	1.01	1.65	3.89	1.77	1.73	3.01	1.97	3.28	3.55
781	C	0.9	0.91	1.10	4.89	0.90	1.98	5.41	1.52	2.17	5.37	2.31	5.40	5.96
782	A	0.9	1.01	0.93	4.11	0.88	3.73	3.70	1.93	3.51	4.47	3.19	3.77	4.91
783	C	0.9	0.64	0.72	3.10	0.63	1.61	3.15	0.87	1.70	3.15	1.60	3.45	3.39
784	G	0.9	1.75	2.55	1.78	1.90	4.18	2.64	3.48	6.66	1.99	5.66	2.02	2.66
785	C	0.9	0.75	1.73	3.12	0.71	1.33	4.04	0.75	1.58	4.06	1.35	3.17	3.09
786	C	1.0	0.89	1.51	2.88	0.96	1.45	4.14	1.01	3.39	3.20	1.76	3.27	2.46
787	G	0.9	0.95	1.43	1.82	0.99	2.69	3.10	1.86	2.50	1.88	2.75	2.63	2.42
788	G	0.9	0.58	4.25	1.49	0.58	5.18	1.63	0.60	1.09	0.89	1.08	1.48	2.80
789	C	0.9	0.79	3.24	6.00	0.95	1.77	5.30	1.25	1.93	4.37	1.71	5.45	3.55
790	T	0.9	0.80	0.79	2.79	0.73	2.21	3.64	1.01	2.02	3.49	2.07	3.33	2.59
791	G	0.7	0.71	0.78	1.83	0.61	1.51	3.09	1.08	1.65	2.55	1.71	2.35	2.30
792	G	0.7	0.68	7.62	1.90	0.70	2.00	2.39	1.23	1.53	2.29	1.09	1.41	1.93
793	C	0.7	0.96	3.00	4.96	1.04	1.64	5.22	1.55	2.47	4.44	2.46	5.26	5.93
794	T	0.9	3.62	3.15	4.68	3.48	8.44	4.42	6.36	10.06	6.06	9.98	5.60	9.26
795	C	0.9	0.74	1.15	3.84	0.83	2.51	5.38	1.37	2.27	4.70	2.13	4.04	4.98
796	G	0.7	1.47	3.80	3.11	1.47	3.96	4.01	2.39	4.61	3.57	4.28	3.33	4.08
797	G	0.7	0.64	4.03	2.41	0.59	1.24	2.10	1.31	0.97	2.16	0.79	1.69	1.87
798	C	0.9	0.88	2.40	6.75	1.05	2.36	8.26	1.38	2.52	7.75	2.45	7.33	7.65
799	T	0.9	1.39	1.53	6.38	1.47	4.56	6.88	2.12	5.80	6.55	5.37	7.58	7.78
800	G	0.9	1.01	1.26	5.25	1.12	3.33	6.31	1.58	3.78	5.26	3.64	4.37	5.09
801	G	0.9	0.67	2.10	3.25	0.67	1.73	99.03	1.17	1.55	3.72	1.76	3.34	1.52
802	C	0.9	1.89	3.85	8.44	1.99	4.01	8.83	2.13	4.35	8.61	4.42	6.34	4.85
803	C	1.0	0.67	0.92	8.84	0.76	2.51	8.78	1.07	2.79	8.54	2.85	9.41	5.59
804	C	1.0	1.83	2.84	8.27	1.64	3.47	10.77	3.17	3.91	7.07	4.07	7.22	5.56
805	G	0.9	5.05	7.67	8.09	6.18	11.32	9.58	6.27	12.22	7.48	10.90	10.99	9.03
806	C	0.7	1.31	2.48	4.58	1.43	3.14	6.07	2.09	3.77	4.52	3.65	5.47	4.94
807	G	0.7	1.55	1.66	4.01	1.47	4.29	4.80	2.29	3.71	4.13	3.51	4.01	2.57
808	A	0.7	1.02	0.85	5.98	1.09	2.53	5.87	0.63	2.88	6.33	2.89	5.46	5.73
809	A	0.6	1.57	1.86	6.85	1.65	6.45	7.24	3.91	5.58	6.28	5.95	7.51	6.04
810	C	0.6	4.01	3.88	5.18	3.65	2.91	5.23	2.02	2.40	4.71	2.43	4.22	2.73
811	C	0.4	0.59	0.82	2.17	0.61	1.98	3.90	0.72	1.39	3.43	1.62	3.09	2.01
812	T	0.6	1.71	1.41	2.73	1.35	4.03	3.56	1.25	2.89	3.60	3.19	3.68	2.43
813	G	0.7	0.96	1.14	5.24	0.94	2.41	4.52	1.18	2.33	4.51	2.54	2.81	3.24
814	T	0.7	1.20	2.32	5.92	1.25	3.97	5.59	2.06	3.38	5.90	3.75	5.00	5.52
815	C	0.7	0.79	0.91	1.79	0.68	2.14	2.80	1.02	1.56	1.76	1.51	2.83	3.21
816	G	0.9	4.90	10.68	8.82	4.90	13.64	8.68	6.59	8.36	8.20	8.33	8.86	7.80
817	C	0.9	0.70	1.21	1.69	0.67	1.35	1.63	0.59	1.39	2.22	1.27	2.28	2.24
818	C	1.0	0.59	1.07	1.89	0.63	2.31	2.25	1.84	1.49	3.03	1.54	3.10	2.16
819	C	1.0	2.90	2.12	2.71	1.54	2.07	5.80	2.86	2.45	2.70	2.76	2.44	2.30
820	G	1.0	3.32	4.46	5.70	2.98	8.84	6.02	4.24	6.69	5.70	7.21	7.27	7.44
821	C	1.0	1.06	2.68	2.34	0.87	1.56	1.93	1.09	1.86	1.67	1.30	1.84	2.74
822	C	1.0	1.17	1.55	1.80	0.91	1.42	3.83	1.28	1.52	2.58	2.34	1.59	1.47
823	G	1.0	3.64	4.10	4.62	3.22	7.25	5.67	3.62	5.08	3.80	4.67	4.91	3.28
824	C	0.9	2.10	1.54	1.73	0.98	1.25	1.96	1.54	1.39	1.60	1.51	1.79	1.95
825	G	0.9	0.91	2.30	3.44	0.97	2.26	3.12	1.60	1.76	3.11	1.87	2.98	2.43
826	C	0.9	0.85	4.04	2.31	0.95	1.26	2.57	1.03	1.52	2.84	1.36	1.91	2.43
827	A	0.7	2.70	2.40	2.95	2.54	4.83	2.72	3.16	3.46	3.88	3.38	3.93	5.07
828	G	0.7	0.60	1.16	3.34	0.72	1.52	3.51	0.97	1.30	2.96	1.24	3.29	2.49
829	C	0.6	0.51	1.10	1.85	0.58	1.29	1.55	1.08	1.14	1.37	1.13	1.08	1.34
830	T	0.4	5.52	5.03	4.69	5.35	10.02	4.47	6.74	5.94	4.99	5.85	4.97	4.83

0 — 1
GC content

0.5 — 2.5
Mutation frequency (kb^{-1})

Fig. S2. Sequence dependency of mutation frequency (enlarged view of Fig. S1, positions 770–830).

Template (pg)			50			100			250			500		
Mn^{2+} (mM)			0	1	2	0	1	2	0	1	2	0	1	2
1000	C	0.7	0.72	1.54	2.19	0.65	1.21	2.47	0.75	1.22	1.77	1.33	2.31	1.45
1001	A	0.7	0.54	0.50	1.83	0.55	1.68	1.70	0.69	1.39	1.50	1.34	2.28	2.19
1002	G	0.9	0.68	1.32	1.82	0.66	1.52	2.96	1.02	1.38	1.95	1.82	0.87	2.49
1003	G	0.9	1.43	1.78	1.52	1.07	1.56	2.26	1.07	1.61	1.45	1.39	1.53	1.94
1004	C	0.9	0.90	2.47	2.56	1.07	1.56	3.57	1.17	1.65	1.71	1.68	1.94	2.24
1005	G	1.0	1.25	2.24	2.04	1.37	1.90	4.55	2.73	2.30	2.79	2.79	2.02	1.88
1006	G	0.9	0.80	1.32	1.59	0.77	1.56	2.65	0.97	1.70	1.75	1.78	1.94	1.54
1007	G	0.9	0.71	2.11	1.71	0.90	1.74	1.94	1.83	1.54	1.69	1.32	1.60	1.98
1008	C	0.9	1.25	4.09	4.27	1.32	2.44	4.46	1.58	2.45	3.57	2.20	4.02	3.20
1009	T	0.9	0.62	0.79	2.53	0.74	2.73	2.21	1.52	2.43	2.73	2.71	2.24	2.58
1010	G	0.7	1.23	1.69	2.49	1.13	2.27	4.94	2.11	2.66	3.25	3.60	3.90	3.71
1011	G	0.7	0.76	1.13	1.76	0.73	1.47	3.51	1.25	1.79	2.58	1.89	1.57	2.63
1012	G	0.7	0.52	0.80	1.63	0.73	1.65	2.18	0.67	1.33	2.30	2.09	1.53	1.76
1013	A	0.9	1.07	2.05	3.59	1.11	5.08	3.72	1.98	3.46	3.87	3.90	3.04	3.75
1014	C	0.9	0.74	3.06	2.14	2.07	2.48	2.60	1.41	1.52	2.04	2.04	2.51	2.49
1015	G	0.7	1.29	1.78	2.43	1.57	3.71	3.17	2.06	3.96	2.93	3.78	3.05	4.08
1016	C	0.7	1.01	1.67	2.32	1.02	2.25	2.90	1.19	2.49	3.30	2.22	2.50	2.97
1017	G	0.9	1.53	2.27	3.00	1.74	4.84	4.47	3.49	5.21	3.96	4.76	4.90	4.53
1018	A	0.7	1.50	1.57	6.21	1.40	7.27	5.83	4.05	5.82	6.00	5.72	6.23	6.18
1019	C	0.6	0.61	1.21	2.00	0.75	2.03	2.65	0.95	2.00	2.42	2.01	3.03	1.91
1020	C	0.6	0.84	1.02	2.12	0.87	2.04	3.69	1.45	2.24	3.43	2.41	2.81	1.56
1021	T	0.4	0.53	0.63	1.95	0.61	1.94	1.84	0.76	1.79	1.99	1.74	2.47	2.51
1022	T	0.4	0.59	0.48	1.86	0.47	1.86	1.94	0.54	1.18	2.04	1.47	1.97	1.82
1023	C	0.4	0.67	0.82	2.73	0.68	1.43	2.93	0.95	1.79	2.68	2.43	3.09	2.03
1024	A	0.4	0.84	1.03	2.16	0.90	2.32	2.00	1.81	2.32	2.78	2.62	2.33	2.23
1025	T	0.4	0.65	0.70	2.94	0.66	2.66	2.88	0.90	1.92	2.86	2.41	2.58	2.83
1026	C	0.6	0.63	0.75	1.67	0.54	1.29	1.80	0.71	1.23	2.13	1.29	2.56	2.41
1027	G	0.6	0.77	1.12	2.44	0.81	2.52	2.33	1.42	2.57	2.52	2.51	3.21	2.51
1028	T	0.6	1.00	1.16	3.79	1.05	4.74	3.91	2.08	4.10	3.88	4.34	3.75	5.34
1029	C	0.7	1.14	1.03	1.70	0.88	1.92	2.70	0.76	1.66	1.80	1.63	3.18	2.38
1030	G	0.7	1.14	1.91	2.93	1.23	3.85	3.74	2.11	3.97	2.88	4.39	4.80	2.98
1031	A	0.6	1.00	1.13	4.24	1.09	4.88	4.37	2.46	4.47	4.94	4.51	3.39	6.28
1032	C	0.7	0.55	1.09	2.03	0.51	1.42	2.65	0.51	1.15	1.89	1.54	1.13	1.89
1033	C	0.7	0.99	1.75	2.58	0.73	1.82	3.69	1.47	2.02	2.97	2.01	2.65	1.75
1034	A	0.7	0.60	1.28	2.30	0.62	2.10	1.97	0.87	1.91	2.16	1.81	2.17	3.12
1035	G	0.9	1.65	2.92	4.31	1.60	3.57	6.13	2.93	3.92	4.14	4.41	4.06	3.28
1036	G	0.9	1.25	1.26	3.99	1.20	2.49	5.43	1.40	2.59	3.58	2.98	2.80	3.18
1037	G	0.9	0.93	3.92	3.47	0.99	2.87	4.34	1.51	2.23	2.72	2.34	4.12	2.21
1038	C	1.0	1.16	4.79	2.63	1.13	2.24	4.09	1.44	2.08	3.24	2.97	2.02	2.70
1039	C	0.9	0.94	1.22	1.85	0.85	1.74	2.75	1.20	1.71	2.32	1.92	2.38	1.81
1040	G	0.9	1.01	5.82	3.24	0.96	3.06	4.20	7.33	3.15	2.73	3.48	3.68	3.20
1041	C	0.9	1.81	2.07	3.41	1.70	2.82	3.46	2.48	3.28	3.32	3.36	3.09	3.05
1042	T	0.9	1.96	2.22	7.11	2.34	9.63	7.14	7.43	9.52	7.11	10.29	9.29	7.97
1043	C	0.9	0.80	1.18	3.06	0.94	2.61	3.97	1.31	2.57	2.16	3.01	3.78	3.11
1044	C	0.7	1.20	1.80	3.06	1.16	2.47	4.47	2.64	3.15	2.59	4.19	2.67	3.54
1045	G	0.7	0.77	1.39	2.41	0.92	2.23	3.86	1.43	2.33	2.10	2.32	2.02	2.85
1046	G	0.7	0.46	1.14	1.54	0.50	1.30	1.91	0.43	1.14	1.51	2.08	1.64	1.53
1047	T	0.7	1.72	1.96	5.27	1.87	7.62	6.01	6.03	6.10	5.91	6.29	5.67	5.81
1048	G	0.7	0.49	0.58	2.18	0.45	0.89	4.28	0.61	1.19	2.25	1.23	1.89	1.26
1049	T	0.6	1.02	1.38	4.25	1.11	5.55	4.56	3.30	4.57	4.06	4.91	4.19	4.34
1050	G	0.4	0.55	0.81	1.83	0.59	1.25	1.62	0.70	1.32	1.52	1.20	2.08	1.25

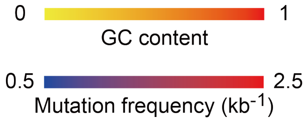

Fig. S3. Sequence dependency of mutation frequency (enlarged view of Fig. S1, positions 1000–1050).

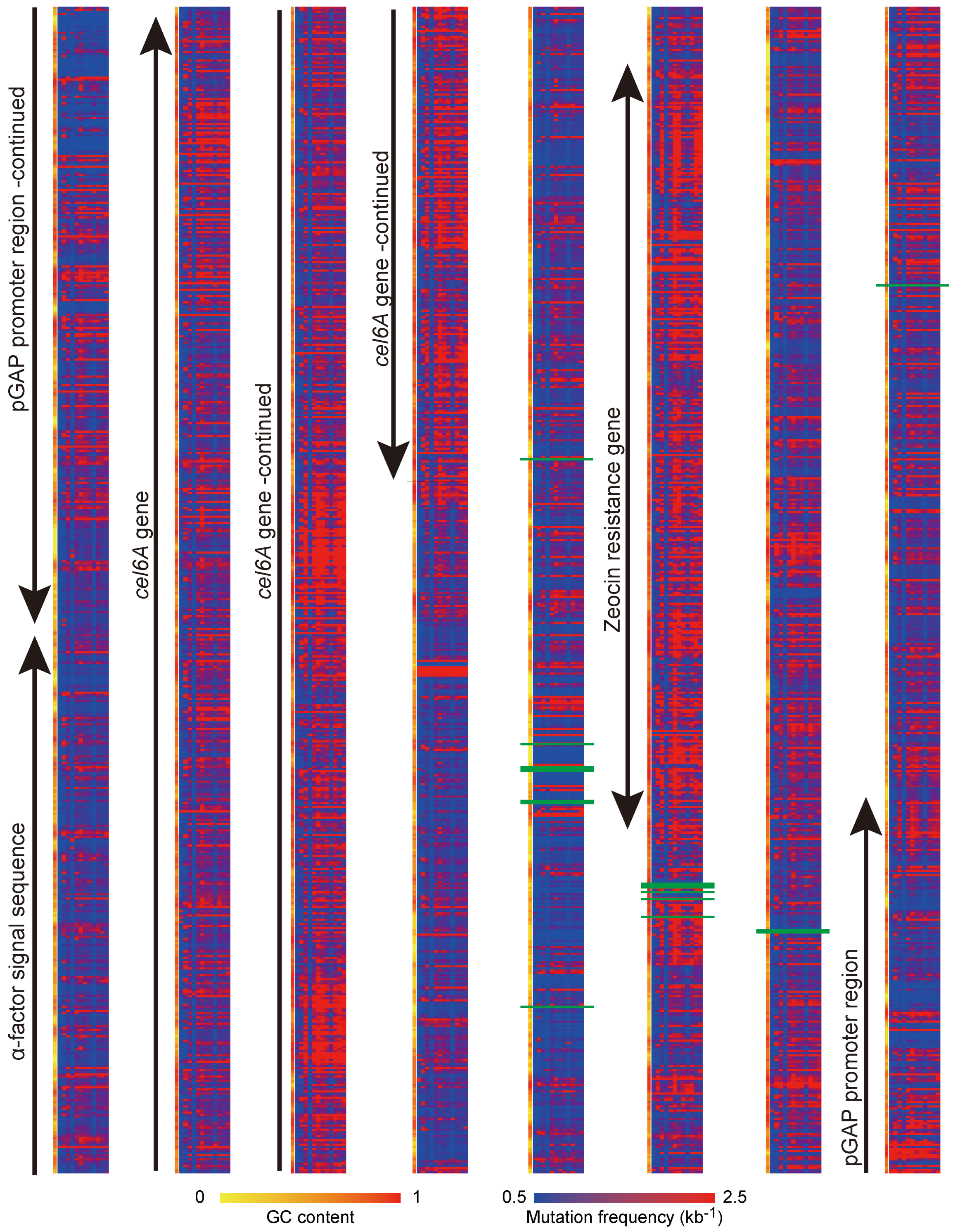

Fig. S4. Sequence dependency of mutation frequency (pGAPZα/*cel6A*). The start position of the sequence is the restriction site of BlnI. The mutations whose mutation rate was almost 99.9% were found in vector regions and indicated by green lines as unrelated mutations.

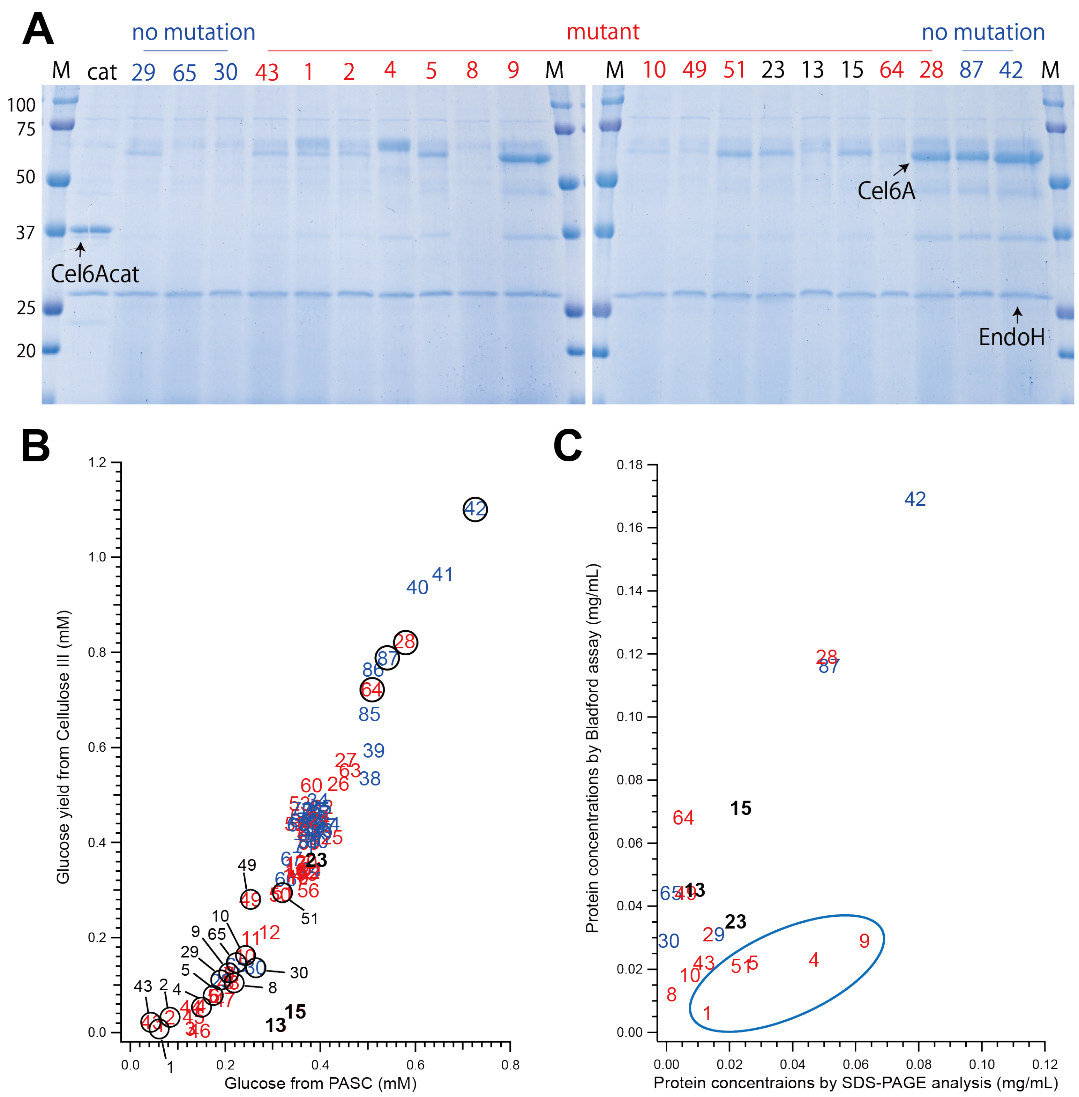

Fig. S5. Protein expression levels of transformants. A) SDS-PAGE of 25 μl culture supernatants after the digestion with endoglycosidase H and α-mannosidase. The catalytic domain of *Pc*Cel6A expressed with *GAP* promoter was used as a control. B) Mutants analyzed by SDS-PAGE indicated in Fig. 5A. C) The protein concentrations calculated from SDS-PAGE analysis and the total protein concentrations of culture supernatants measured by Bradford assay with BSA as a standard. The protein concentrations estimated by Bradford assay well reflect that estimated from SDS-PAGE analysis at the higher protein concentrations, while the correlation at low productivity level is ambiguous. The concentrations of some crude enzymes were underestimated in Bradford assay compared with SDS-PAGE (circled by blue).
